# Ultra-low dose infection imaging of a newborn without sedation using long axial field-of-view PET/CT

**DOI:** 10.1007/s00259-022-05979-3

**Published:** 2022-09-27

**Authors:** N. D. van Rijsewijk, B. van Leer, O. V. Ivashchenko, E. H. Schölvinck, F. van den Heuvel, J. H. van Snick, R. H. J. A. Slart, W. Noordzij, A. W. J. M. Glaudemans

**Affiliations:** 1 Medical Imaging Center, Department of Nuclear Medicine and Molecular Imaging, University Medical Center Groningen, University of Groningen, Groningen, the Netherlands; 2 Department of Critical Care, University Medical Center Groningen, University of Groningen, Groningen, the Netherlands; 3 Department of Pediatric Infectious Diseases, University Medical Center Groningen, University of Groningen, Groningen, the Netherlands; 4 Department of Pediatric Cardiology, Beatrix Children’s Hospital, University Medical Center Groningen, University of Groningen, Groningen, the Netherlands

  A 10-week-old newborn presenting with *Staphylococcus aureus* sepsis of unknown origin was referred to our nuclear medicine department for ^18^F-FDG PET/CT imaging. His medical history consisted of several congenital defects, including Fallot’s tetralogy and jejunal atresia. A total body ^18^F-FDG PET/CT imaging was performed in 3 min (1-bed position) without sedation on the Biograph Vision Quadra system (Siemens Healthineers, Erlangen, Germany) using only 12 MBq of activity, representing less than half of the minimum activity recommended by the EANM dose chart [1]. Effective patient dose was 1.3 mSv for internal radiation (PET) [2], while external radiation from low-dose CT contributed an additional 0.65 mSv [3]. Values remained at least two times below international recommendations [1, 4].

The scan showed normal physiological uptake in the kidneys, bladder, myocardium, and developing brain, including physiological hypometabolism in the frontal cortex (A). The intense ^18^F-FDG uptake in the mouth region is due to the use of a pacifier (A, B). Two infectious foci were detected: the first at the dorsum of the right foot (A and B: green arrow) around the intravenous access line and the second at the bottom of the right atrium near the entrance of the inferior caval vein (C: yellow arrow). Reactive bone marrow uptake is also visible (A). Ultrasound of the foot revealed a subcutaneous abscess, which was drained; culturing detected *S. aureus*. The ^18^F-FDG uptake in the heart corresponded with a solid, echodense structure suggestive of a thrombus or vegetation. This is seen on transthoracic echocardiography (D: blue arrow) at the eustachian valve (usually absent by adulthood), indicative of possible endocarditis. The patient improved under antibiotic therapy.

This case confirms the power to image infections at such a young age by using ultrafast long axial field-of-view (LAFOV) PET/CT with ultra-low dose administered activity without the need for sedation [5].
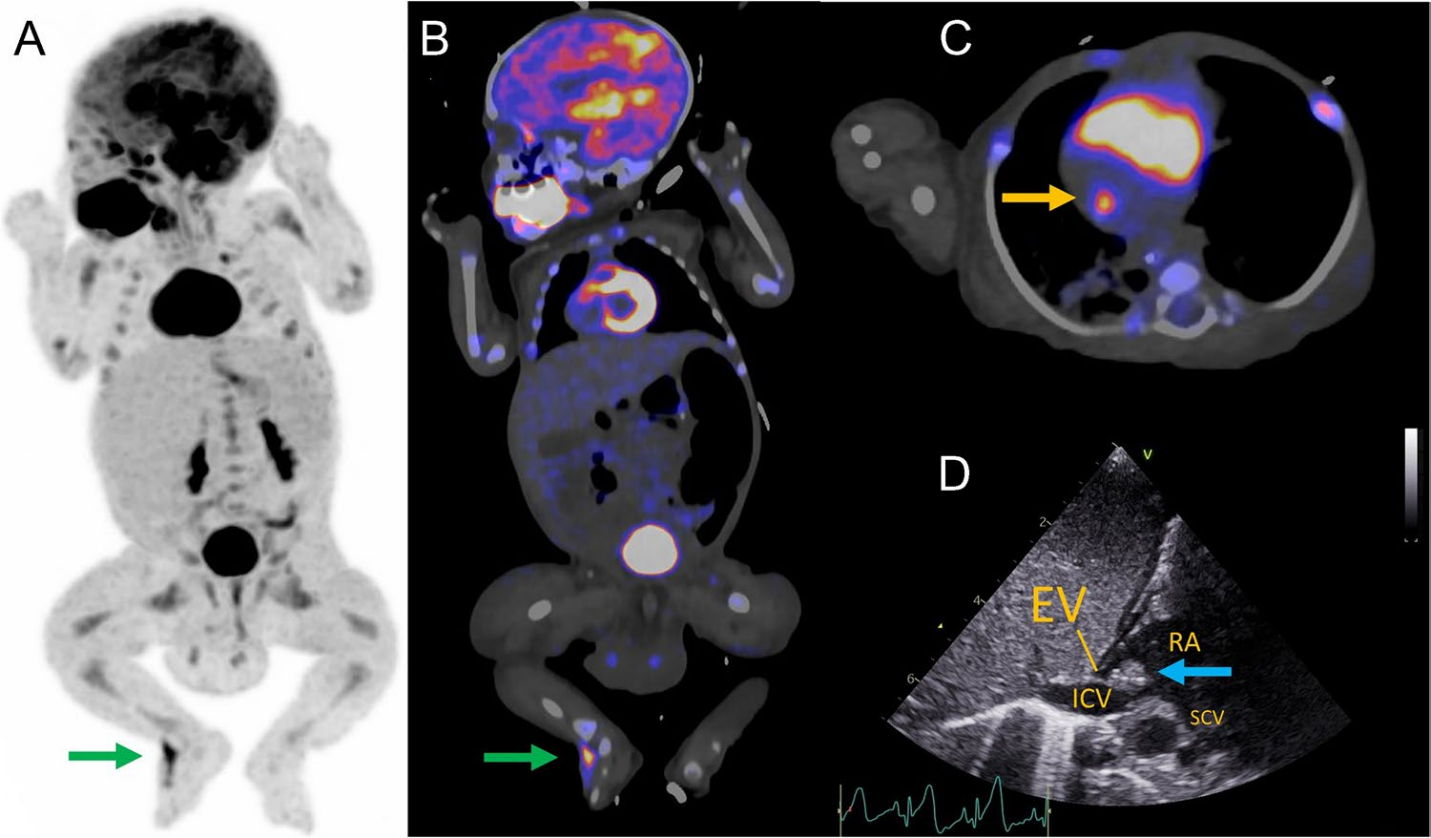


## Abbreviations

EV: Eustachian valve
ICV: Inferior caval vein
RA: Right atrium
SCV: Superior caval vein

## References

[1] EANM Dose Card (version 5.7.2016) [Internet]. [cited 2022 Aug 2]. Available from: https://www.eanm.org/initiatives/dosage-card/

[2] ICRP (2008). Radiation dose to patients from radiopharmaceuticals Addendum 3 to ICRP Publication 53. ICRP Publication 106. Ann ICRP.

[3] Valentin J, International Commission on Radiation Protection (2007). Managing patient dose in multi-detector computed tomography(MDCT). ICRP Publication 102. Ann ICRP.

[4] European Commission. Directorate General for Energy. European guidelines on diagnostic reference levels for paediatric imaging. [Internet]. LU: Publications Office; 2018 [cited 2022 Aug 3]. Available from: https://data.europa.eu/doi/10.2833/003998

[5] Slart RHJA, Tsoumpas C, Glaudemans AWJM, Noordzij W, Willemsen ATM, Borra RJH (2021). Long axial field of view PET scanners: a road map to implementation and new possibilities. Eur J Nucl Med Mol Imaging.

